# Analysis of Anxiety Levels of Nursing Students Because of e-Learning during the COVID-19 Pandemic

**DOI:** 10.3390/healthcare9030252

**Published:** 2021-03-01

**Authors:** Jessica García-González, Wei Ruqiong, Raquel Alarcon-Rodriguez, Mar Requena-Mullor, Can Ding, Maria Isabel Ventura-Miranda

**Affiliations:** 1 Department of Nursing, Faculty of Health and Social Sciences, Campus de Lorca, University of Murcia, 30800 Murcia, Spain; jessyka_gg@um.es; 2 Department of Rehabilitation Medicine, The First Affilited Hospital of Guangxi Medical University, Nanning 530021, China; weiruqiongxibanya@163.com; 3 Department of Nursing, Physiotherapy and Medicine, The University of Almería, 04120 Almería, Spain; ralarcon@ual.es (R.A.-R.); mvm737@ual.es (M.I.V.-M.); 4 West China School of Basic Medical Sciences and Forensic Medicine, Sichuan University, Chengdu 610017, China; erin.ding@zailaboratory.com

**Keywords:** nursing students, COVID-19, state-trait anxiety, mental health, pandemic, confinement

## Abstract

The continued expand of the coronavirus disease 19 (COVID-19) pandemic, confinement measures and an urgent change in the education of nursing students from traditional education to distance learning throughout the country affect the mental health of university students. This study analyzed state-trait anxiety levels of nursing students because of e-Learning during two periods of the COVID-19 confinement. A mixed follow-up study was used, which evaluates the same cohort cross-sectionally but with a longitudinal component. A sample of 460 nursing students of the Nursing Degree at the University of Lorca (Murcia) and University of Almería (Spain) completed an online anxiety level questionnaire using the Spielberger State-Trait Anxiety Inventory (STAI) during the first and fourth weeks of the COVID-19 confinement. Total anxiety levels increased in the fourth week, as compared to the first week (first week: 50.4 ± 20.8 and fourth week: 59.9 ± 10.6 (*p* < 0.001)). The linear regression model showed that the significant predictors for STAI anxiety in nursing students were being in the last year of the degree program, being female, and spending confinement in a house without a garden. In general, most nursing students were emotionally affected by high levels of anxiety of state-trait during the COVID-19 outbreak.

## 1. Introduction

The World Health Organization (WHO) declared the coronavirus disease 19 (COVID-19) outbreak as a Public Health Emergency of International Concern and considered the outbreak as a global pandemic [1]. In addition to a risk of death by infection, the epidemic has placed significant psychological stress on people around the world [2,3].

At a global level and at high speed, this situation has caused the closure of schools and universities, affecting more than 1.57 billion students in 191 countries [4]. The training of nursing students has been modified, for which multiple educational solutions have been deployed, all of them based on distance education. Teachers who are knowledgeable about the didactics of traditional classes have been forced to tackle e-learning overnight, although not all were well prepared. The same has happened with students, they have gone from a model based on face-to-face learning, to a model in which students must freely and voluntarily engage in their distance learning [5]. At the international level, a great variety of learning and assessment criteria have appeared that are adapted to their national, work, and social contexts [6]. In Spain, the Association of Spanish Universities (CRUE) has recommended adapting the assessment tests using distance assessment procedures [7].

A study carried out on medical students from Singapore, on teaching through Zoom, described the overall satisfaction of students [8]. A qualitative study carried out in Spain on 32 nursing students, who switched from face-to-face learning to e-learning during the pandemic, confirmed that e-learning was more worrying for older students, those from rural areas, with job responsibilities and relatives, and students with limited electronic resources [9]. Another qualitative study of 60 medical students from Saudi Arabia indicated that online learning was well received and that students recognized some advantages of such education, such as saving time and greater usefulness of time. However, the students indicated that they also encountered multiple challenges, including methodological problems, problems with perceptions of content, and technical and behavioral problems during educational sessions and online examinations [10]. A recent study carried out in health sciences university student’s in Croatia during the COVID-19 pandemic highlights that most students were satisfied with the exclusive e-learning, as well as with their personal and institutional adaptation to it [11].

On the other hand, anxiety and stress have been recognized as important issues experienced by nursing students throughout their education [12,13]. Chernomas and Shapiro showed that nursing education has consistently been associated with anxiety among students: heavy course loads, continued pressure to attain a high grade point average, stringent examinations, complex interpersonal relationships, and challenges of the clinical environment [14]. Outbreaks can create significant psychological stress that can lead to adverse effects on students’ learning and overall psychological health. There are studies that research post-outbreak stress, which may not reflect the actual stress subjects felt during the event itself [15]. In the study conducted on university students in Lebanon, they concluded that the sudden change that occurred during the pandemic has impeded students from learning and caused stressful workloads that began to lead to anxiety and depressive symptoms [16]. There are a few studies that analyze the level of anxiety only of the university nursing degree students during the COVID-19 outbreak. Some studies were performed on health sciences students [11,17] and in other disciplines [15,18,19,20].

There is also a research about the psychological impact of the epidemic on the general population, patients, health workers, children, and older adults [21,22]. However, a few studies analyzed the level of anxiety of university nursing students during the COVID-19 outbreak, and there were also the studies that were performed on medical students [10,17,18]. Therefore, the objective of our study was to analyze state-trait anxiety levels of nursing students because of e-Learning during two different periods throughout the confinement caused by COVID-19.

## 2. Materials and Methods

### 2.1. Study Design

A mixed follow-up study was used, which evaluated the same cohort cross-sectionally but with a longitudinal component. The study cohort was evaluated in two different time periods. It was conducted on a cohort of 460 individuals aged 18–25 years.

### 2.2. Participants and Selection Criteria

The study population consisted of a total of 460 nursing students of the Nursing Degree at the University of Lorca (Murcia) and University of Almería (Spain). This study was conducted over two periods during the confinement for the COVID-19 outbreak: the first week of confinement and the fourth week of confinement. In both periods, the participants completed an online questionnaire on state-trait anxiety levels motivated by the change in the teaching modality, going from a face-to-face model to e-learning.

During the first period of the study, the number of participants amounted to 510 individuals, with 28 participants being excluded from the study for not having completed the questionnaire correctly, leaving a total of 482 participants. During the second period, of these 482 individuals, 14 individuals failed to respond and a further 8 individuals failed to complete the whole questionnaire. As a result, these individuals were excluded from the study, bringing the final number of participants to 460.

The inclusion criteria were as follows: (1) individuals over 18 years of age, (2) who completed the questionnaire both during first week (from 15 to 22 March 2020) and fourth weeks (from 12 to 19 April 2020) of confinement for the COVID-19 outbreak, (3) with no previous mental health problems, and (4) inscribed in a nursing undergraduate program during COVID-19 confinement. The exclusion criteria were the following: (1) individuals under 18 years of age, (2) who failed to complete the whole questionnaire during the first week and the fourth week of the COVID-19 confinement (3) with previous mental conditions, (4) individuals who only participated in the first period of the study, and (5) not inscribed in a nursing undergraduate program during COVID-19 confinement.

The recruitment of students was carried out through talks in the virtual classroom, where the purpose, procedures, and questionnaires of the study were explained. The Participant Information Sheet (PIS) was incorporated into the virtual classroom, where the most important items of the study were displayed.

### 2.3. Outcome Measures

In this study, the Spielberger State-Trait Anxiety Inventory (STAI) questionnaire was used for self-assessment [23,24]. The online questionnaire was sent to the students through an online questionnaire platform e-learning. Participants were able to complete the questionnaire on their computer or mobile phone, where they could access it through a link to the platform e-learning. The online survey was distributed along with an invitation email containing information on the purpose of the study and data anonymity and confidentiality. In the questionnaire participants were told that they gave their consent when filling it out. The self-assessment questionnaire was completed by participants voluntarily, according to standardized guidance methods. The variables included in the questionnaire were as follows:

- Socio-demographic variables: sex, age, academic course, and university where study.
- Confinement-related variables: type of housing, number of times outdoors, number of individuals living together at home during the confinement, and work situation of your parents (during confinement).
- The STAI questionnaire: State-Trait Anxiety Inventory (STAI). This questionnaire was developed by Spielberger et al. in 1970 [23] and adapted to Spanish in 2015. The Spanish questionnaire was validated with a Cronbach’s alpha of 0.9 for the Anxiety trait and 0.94 for the Anxiety state, which were very similar to the original questionnaire [24]. A 40-item self-report questionnaire assessing two subscales, i.e., anxiety as state (AS), which assesses the transient emotional state “at this moment,” the scale consisting of 20 items, and the anxiety as trait subscale (AT), which analyses the relatively stable proneness to anxiety, in general, was used; this scale also consisted of 20 items. Both subscales use a 4-point Likert scale (0 “almost never/not at all”; 1 “sometimes /somewhat”; 2 “often/moderately so”; 3 “almost always/very much so”). The total score for each of the subscales ranges from 0 to 60 points. The subscales include both positive and negative anxiety items. Anxiety levels are classified as follows: state anxiety and trait anxiety [25].

### 2.4. Ethical Considerations

The online survey was distributed along with an invitation email containing information on the purpose of the study and data anonymity and confidentiality. In the questionnaire participants were told that they gave their consent when filling it out. The study was conducted according to the guidelines of the Declaration of Helsinki and approved by the Ethics Committee of University of Murcia (ID: 2982/2020).

### 2.5. Data Analysis

A dedicated database was created to store collected data. The data were analyzed using the statistical software IBM SPSS version 26.0 (IBM SPSS, Chicago, IL, USA). A descriptive analysis was carried out, the means and standard deviations (SD) were calculated for continuous variables, and the absolute and relative frequency distributions were calculated for categorical variables. Normal distribution was assessed by the Kolmogorov–Smirnov test. The Student’s *t*-test and the ANOVA test were utilized for the comparison of independent variables. The related variables were explored using Student’s *t*-test. Hedges’s G was used to determine the size of the effect. The Chi-square test was utilized for comparison, and categorical variables were expressed as frequencies and percentages. For the multivariate analysis, multiple linear regression was performed. A *p*-value of <0.05 was considered to be statistically significant.

## 3. Results

A total of 460 nursing students participated on the influence of the COVID-19 pandemic on anxiety levels during confinement.

### 3.1. Sociodemographic Variables of Nursing Students

The average age of the participants was 20.58 ± 1.54 years old. Among females, the average age was 20.59 ± 1.53 years old and among males, 20.54 ± 1.60 years old, without statistically significant differences (*p* = 0.78) (Table 1).

In this study, 78% of the participants were female (*n* = 359) and 22% male (*n* = 101). Moreover, 23.9% of the nursing students were in the first year of their degree, 25.9% in the second year, 23% in the third year, and 27.2% in the last year of their course of study. Similar percentages resulted when analyzing the distribution of students in their year of study based on sex, with no statistically significant differences (*p* = 0.78).

The average number of people confined to the participants’ homes was 3.75 ± 1.52, and the majority lived in houses with gardens (60.4%). Statistically significant differences were not observed when comparing males and females, both in the average number of confined people and the type of housing where they were spending the confinement.

Additionally, 39.8% of the students had not left the house during the confinement, while 8.9% left the house every day. The majority of females (77.7%) did not leave the house or did so only 1 or 2 times per week, and in the case of males, to a lesser extent (51.5%). The percentage of males (12.9%) who left the house every day during lockdown was higher than the percentages of females who did the same (7.8%). Upon comparing the number of times that the participants left the house by sex, statistically significant differences were observed (*p* < 0.001).

Regarding the employment status of the students’ parents during the COVID-19 confinement, 58% of the parents were unemployed, while 42% of them continued working. Similar percentages were observed when analyzing the results by sex, where 58.2% of the parents of female students were unemployed, while 57.4% of the parents of male students were unemployed (*p* = 0.88). All of the students tested negative for COVID-19.

### 3.2. State-Trait Anxiety Differences between the First and Fourth Week of Confinement of COVID-19

Table 2 compares the average scores of the STAI of the study variables in the first week and the fourth week of confinement. Table 3 compares the average values of the STAI between the first and fourth weeks of confinement, the change in scores within each variable with the confidence interval (CI) of 95%, and Hedges’s G coefficient.

In the fourth week of confinement, STAI scores were higher than the first week of being confined in both males and females. Although female students had higher anxiety levels than the male students in both weeks, the increase in males’ anxiety level was higher than in females. These results are statistically significant.

During the first and fourth week of confinement, the students of the different years in their degree program had similar STAI scores, without statistically significant differences between them in either week. All students’ STAI scores increased throughout confinement, with undergraduate students in their last year of their degree program showing the highest increase in anxiety levels in the fourth week with respect to the first week of confinement (*p* < 0.001).

Anxiety levels in students in confinement in a house with a garden were lower than those living in a house without a garden throughout the confinement, with statistically significant differences observed. The increase in anxiety levels observed in the fourth week, as compared to the first week, was similar in people living in a home with a garden and a home without a garden.

Students who did not leave the house, who went out a few days a week, or those who went out every day showed similar average scores on the STAI in the first week of confinement (*p* = 0.07). After four weeks of confinement, their anxiety levels increased, regardless of how many times a week they left the house, although the students who had not left the house at all since the beginning of confinement had a greater increase in anxiety levels (*p* < 0.001).

Concerning the employment status of parents, in the first week, as well as the fourth week of confinement, the average scores on the STAI were similar between the students whose parents were unemployed and those whose parents continued working, without statistically significant differences. Both scores increased throughout the period of confinement, with a greater increase in anxiety levels among students whose parents were unemployed due to the COVID-19 pandemic (*p* < 0.001).

### 3.3. Score of STAI-T, STAI-S, and STAI Total

When analyzing the anxiety levels in nursing students, it was observed that during the first week of confinement, the score of state anxiety was 26.5 ± 11.7, increasing to a value of 31.1 ± 6.9 in the fourth week of confinement. The STAI score for trait anxiety in the first week was 23.9 ± 10.7, and in the fourth week, increased to 28.7 ± 6.5. The increase in trait anxiety was greater than the increase in state anxiety. Total anxiety levels also increased in the fourth week, as compared to the first week, from an average score of 50.4 ± 20.8 to 59.9 ± 10.6. All these results were statistically significant (*p* < 0.001) (Table 4).

### 3.4. Multiple Linear Regression Model

Table 5 shows the multiple linear regression models, in which the dependent variable is anxiety level (STAI) and the independent variables are sex (female/male), academic course (first/second/third/fourth), and type of housing during confinement (garden/no garden) for nursing students.

The multiple linear regression model was found to meet the conditions of autocorrelation, linearity, collinearity, homoscedasticity, and normality.

The equation that describes the final model was: Anxiety level (STAI) = 41.9 + 7.1 female + 5.0 fourth year of degree + 4.4 housing without a garden.

The linear regression model showed that the significant predictors for STAI anxiety in nursing students were being in the last year of the degree program, being female, and spending confinement in a house without a garden.

## 4. Discussion

The COVID-19 outbreak is likely one of the most challenging threats to national and international public health in recent decades. The pandemic has had a major impact on nearly all sectors, especially on the health sector. Initially, healthcare students were placed in a stressful situation due to uncertainty regarding the transmission of the disease, fear and implementation of rigorous disease control protocols. Similarly, health students, nurses and doctors, were exposed during the outbreak to similar stressors, which could also affect their academic achievements and negatively influence their learning, thus causing them an even higher level of anxiety [17]. During the pandemic, the training of nursing students has been modified, for which multiple educational solutions have been deployed, all of them based on e-learning [5].

Although the digital transition that occurred at the beginning of the pandemic created confusion, e-learning has advantages in quarantine times where students can still benefit from catching up on their classes. However, the reality is that certain topics are much more difficult to offer online, especially those that involve practical or even clinical aspects in health sciences specialties, as is the case with nursing students. Fawaz and Samaha showed in the study carried out on university students in Lebanon that the sudden change that occurred during the pandemic has prevented students from learning and caused stressful workloads that began to give rise to anxiety and depressive symptoms [16].

There are several studies found that showed student satisfaction with the new forms of learning, e-learning, implemented during confinement. In a recently published study of Singapore medical students, Srinivasan, found that students expressed satisfaction when asked about teachings through Zoom [8]. Another study carried out during the pandemic on university students of health sciences in Croatia highlighted that the majority of students were satisfied with the exclusive e-learning, as well as with its adaptation [11]. Khalil et al. conducted a qualitative study with 60 medical students from Saudi Arabia, indicated that online learning was well received and that students recognized some advantages of such education, such as saving time and greater usefulness of the weather [10]. A qualitative study conducted in Spain on 32 nursing students, who switched from face-to-face learning to e-learning during the pandemic, confirmed that e-learning was more worrying for older students, those from rural areas, with work and family responsibilities, and students with limited electronic resources [9].

Anxiety is a common psychological phenomenon in any disaster and can be a barrier to medical and mental health interventions. The public psychological state must be taken into account in order to provide appropriate and quality mental health support [26].

Quarantine is an effective measure to curb the spread of an epidemic, but the protocols established may be difficult to apply [21]. A recently published review by Brooks et al. studied the consequences of quarantine, describing it as an unpleasant experience for those who endure it, mostly due to their separation from the ones they care about, the loss of liberty, not knowing certainly about the state of the disease and boredom. All of this can trigger the onset of mental disorders and health-related symptoms in adolescents and adults. Mental disorders can include anxiety, sadness, insomnia, anger, and feeling insecure [2,27].

In the available literature, we can find studies in which the authors report high levels of anxiety among students in health degrees (nursing and medicine) [19,20,28,29].

In our study, we reported that nursing students in the last year showed a greater increase in anxiety levels in the fourth week of confinement compared to the first week. Chen [30] found that when nursing students experience excessive anxiety, their motivation to study, their effectiveness, and their eagerness to become nurses are negatively impacted, which could explain why the fourth-year nursing students showed the greatest increase in anxiety levels compared to the rest of the students.

### 4.1. Anxiety Levels and COVID-19

A longitudinal study in China, using online questionnaires at two points in time during the COVID-19 outbreak, showed that while stress, anxiety, and depression levels in the general population remained stable at both points, in the age range of 12–21 years old, the levels increased. This age range consists mainly of students who were affected by the closure of colleges and universities [31]. Other authors also informed about a greater rate of anxiety and depression among the population from 21 to 30 years old, with a level of anxiety and depression of 27.3% and 46%, respectively, in the Chinese province of Hubei, during the COVID-19 outbreak [32]. Savitsky et al. recently published a study during the COVID-19 outbreak, on 244 students nursing in the Ashkelon Academic College in Israel. The researchers reported that nursing students has prevalence of moderate and severe anxiety of 42.8% and 13.1%, respectively [33]. These results correspond with those found in this study, in which nursing students reported an increase in anxiety levels in the fourth week of confinement compared to the first week.

Cao et al. recently published a study on 7.143 students in the School of Medicine in Changzhi (China), using structured questionnaires. The researchers concluded that 24.9% of the students expressed having experienced anxiety during the COVID-19 outbreak, i.e., 0.9% have presented severe anxiety and 21.3%, mild anxiety, regardless of their sex. Our findings concluded that females demonstrated higher levels of anxiety than males at both points in time during quarantine. In our study, it was shown that after four weeks of confinement, all the nursing students had increased anxiety levels, regardless of the number of times they left their home. However, the students who showed the greatest increase in anxiety levels were those who had not gone out throughout the entire period they had been confined. In addition, nursing students that lived in homes without gardens reported higher levels of anxiety. The employment status of the students’ parents (employed or unemployed), increased anxiety levels alike in students in both the first and fourth weeks of confinement. The study published by Cao et al. reported that medical students that lived in rural areas, without their parents, had families without stable income, and who had a family member or knew someone infected with COVID-19 were more likely to have high anxiety levels [17].

Wang C et al. conducted 1210 online questionnaires during the first 2 weeks of the COVID-19 outbreak in China. From these questionnaires, they found that 53.8% of the participants rated the psychological impact of the outbreak as moderate or severe; 16.5% of the surveyed reported moderate to severe symptoms of depression; 28.8% reported moderate to severe anxiety symptoms, and 8.1% reported modern to severe stress levels. Moreover, 52.8% of the surveyed participants were students, and they reported feeling a higher level of stress and anxiety compared to those that were employed. A higher level of stress, anxiety, and depression was significantly indicated by males [31]. Our results reported that the nursing students had high levels of anxiety in the first as well as the fourth week of confinement, as well as a significant increase in anxiety levels in the fourth week. Female students had higher anxiety levels than males in both weeks, however, the increase in anxiety levels from the first week to the fourth week was higher in male nursing students.

Araújo et al. highlighted the increase in the levels of depression and anxiety during the outbreak of this coronavirus disease, stemming from uncertainty and information overload. All of this seems to have a negative impact on education, and therefore, on psychological pain and suffering [34]. Reducing students’ anxiety is important in order to promote positive learning outcomes [35]. Nurses generally become nurses out of a desire to help people regain and maintain good health. During the COVID-19 outbreak, there may be a very few occasions to help those who are seriously ill. This inability to save lives will have negative effects both physically and emotionally on the front-line health personnel [36]. Promoting student well-being is an important consideration to bear in mind in order to improve their health status and facilitate the successful progression of their university program with the subsequent transition to professional practice.

### 4.2. Strengths and Limitations

The strength of this study is that we explored the state-trait anxiety level of the nursing student because e-learning during two periods of the confinement prompted by the COVID-19 outbreak. We conducted a multi-center longitudinal study to reflect state-trait anxiety in nursing students and subsequently analyzed the related factors.

There are also some limitations to our research. First, this study used an online questionnaire, which may have introduced some reporting bias. Second, this is a longitudinal study, which would require further active observation over time. A prospective randomized study could better determine correlation and causality.

## 5. Conclusions

Due to the COVID pandemic, most academic institutions around the world have been forced to suspend face-to-face teaching and have adopted the use of e-learning. The sudden shift to exclusive e-learning instructional methods has raised anxiety levels in nursing students, especially those in their last academic year. The highest anxiety levels were associated with being female, being in the last year of the nursing degree, and living in a house without a garden. In general, nursing students were emotionally affected by high levels of state-trait anxiety during COVID-19 confinement.

## Figures and Tables

**Table 1:** Comparison sociodemographic data between women and men in nursing students.

Characteristic		Nursing Students (*n* = 460)	Women (*n* = 359)	Men (*n* = 101)	*p*-Value
Age, in years ^1^		20.58 ± 1.54	20.59 ± 1.53	20.54 ± 1.60	0.78 *
Sex	Male	101 (22%)	–	–	–
	Female	359 (78%)	–	–	
Academic course	First	110 (23.9%)	83 (23.1%)	27 (26.7%)	0.78 **
	Second	119 (25.9%)	92 (25.6%)	27 (26.7%)	
	Third	106 (23%)	86 (24%)	20 (19.8%)	
	Quarter	125 (27.2%)	98 (27.3%)	27 (26.7%)	
Number of people confined ^1^		3.75 ± 1.52	3.81 ± 1.44	3.54 ± 1.77	0.26 *
House type	With a garden	278 (60.4%)	217 (60.4%)	61 (60.4%)	0.99 *
	No garden	182 (39.6%)	142 (39.6%)	40 (39.6%)	
Outings to the street	Never	183 (39.8%)	152 (42.3%)	31 (30.7%)	<0.001 **
	1–2	148 (32.2%)	127 (35.4%)	21 (20.8%)	
	3–5	88 (19.2)	52 (14.5%)	36 (35.6%)	
	All days	41 (8.9%)	28 (7.8%)	13 (12.9%)	
Parents work situation in confinement COVID19	Employed	193 (42%)	150 (41.8%)	43 (42.6%)	0.88 **
	Unemployed	267 (58%)	209 (58.2%)	58 (57.4%)	

^1^ Data are mean ± SD; *p*-value obtained with * Student’s *t*-test. or ** Chi-squared test.

**Table 2:** Comparison of the mean scores for the degree of anxiety in the first and fourth weeks post-confinement of the coronavirus 19 (COVID-19) COVID-19.

Characteristic		STAI 1-Week	*p*-Value	STAI 4-Week	*p*-Value
Sex	Male	44.7 ± 19.4	0.002 ^a^	58.5 ± 10.6	0.14 ^a^
	Female	52.0 ± 20.9		60.3 ± 10.3	
Academic course	First	49.4 ± 22.7	0.15^b^	57.4 ± 21.2	0.04 ^b^
	Second	48.9 ± 21.1		60.8 ± 12.0	
	Third	54.4 ± 20.8		60.8 ± 6.0	
	Quarter	49.3 ± 18.3		60.4 ± 5.0	
House type	With a garden	48.7 ± 20.2	0.03 ^a^	54.4 ± 10.9	<0.001 ^a^
	No garden	53.0 ± 21.3		60.6 ± 10.2	
Outings to the street	Never	45.7 ± 19.8	0.07 ^b^	60.6 ± 10.4	0.83 ^b^
	1–2	50.9 ± 19.1		60.0 ± 8.6	
	3–5	51.2 ± 22.2		59.6 ± 11.6	
	All days	55.1 ± 21.1		59.0 ± 12.8	
Parents work situation in confinement COVID-19	Employed	50.2 ± 20.9	0.79 ^a^	59.1 ± 8.8	0.06 ^a^
	Unemployed	50.7 ± 20.6		61.0 ± 12.6	

Values are expressed as mean ± SD for the first and fourth weeks post-confinement; ^a^ *p*-value obtained with Student’s *t*-test; ^b^ *p*-value obtained with ANOVA test.

**Table 3:** Comparison of the mean scores for the degree of anxiety between first and fourth weeks post-confinement of COVID-19.

Characteristic		STAI 1-week	STAI 4-week	Change Diff in Means (95% CI)	*p*-Value	Hedges’s G
Sex	Male	44.7 ± 19.4	58.5 ± 10.6	−13.8 (−17.6, −9.9)	<0.001 ^a^	−0.71
	Female	52.0 ± 20.9	60.3 ± 10.3	−8.2 (−10.6, −5.8)	<0.001 ^a^	−0.35
Academic course	First	49.4 ± 22.7	57.4 ± 21.2	−8.0 (−13.0, −3.0)	0.002 ^a^	−0.30
	Second	48.9 ± 21.1	60.8 ± 12.0	−11.8 (−15.8, −7.7)	<0.001 ^a^	−0.53
	Third	54.4 ± 20.8	60.8 ± 6.0	−6.4 (−10.7, −2.1)	0.004 ^a^	−0.28
	Quarter	49.3 ± 18.3	60.4 ± 5.0	−11.0 (−14.4, −7.7)	<0.001 ^a^	−0.59
House type	With a garden	48.7 ± 20.2	54.4 ± 10.9	−5.7 (−8.4, −4.0)	0.003 ^a^	−0.31
	No garden	53.0 ± 21.3	60.6 ± 10.2	−7.5 (−10.7, −4.3)	<0.001 ^a^	−0.34
Outings to the street	Never	45.7 ± 19.8	60.6 ± 10.4	−14.9 (−19.2, −10.6)	<0.001 ^a^	−0.73
	1–2	50.9 ± 19.1	60.0 ± 8.6	−9.0 (−12.5, −5.5)	<0.001 ^a^	−0.42
	3–5	51.2 ± 22.2	59.6 ± 11.6	−8.4 (−12.0, −4.8)	<0.001 ^a^	−0.34
	All days	55.1 ± 21.1	59.0 ± 12.8	−3.9 (−10.4, 2.5)	0.22 ^a^	−0.19
Parents work situation in confinement COVID-19	Employed	50.2 ± 20.9	59.1 ± 8.8	−8.9 (−11.5, −6.2)	<0.001 ^a^	−0.40
	Unemployed	50.7 ± 20.6	61.0 ± 12.6	−10.2 (−13.5, −6.9)	<0.001 ^a^	−0.43

Values are expressed as mean ± SD for the first and fourth weeks post-confinement means and as mean (95% CI) for within-group change scores; ^a^ *p*-value obtained with Student’s *t*-test.

**Table 4:** Comparison of the mean scores for the degree of state-trait anxiety in the first and fourth week.

Anxiety	1-Week	4-Week	Change Difference in Means (95% CI)	Hedges’s G	*p*-Value
STAI-T	26.5 ± 11.7	31.1 ± 6.9	−4.6 (−5.8, −3.4)	−0.35	<0.001 *
STAI-S	23.9 ± 10.7	28.7 ± 6.5	−4.7 (−5.9, −3.6)	−0.39	<0.001 *
STAI	50.4 ± 20.8	59.9 ± 10.6	−9.4 (−11.5, −7.4)	−0.41	<0.001 *

*p*-value obtained with * Student’s *t*-test; Results are expressed as mean ± SD.

**Table 5:** Linear regression model predicting anxiety level of nursing students.

Predictors/Co-Variates	Unstandardized Coefficients		Standardized Coefficients	*p* *	95% IC		Co-Linearity Statistics	
	B	Standard Error	Beta		Lower	Upper	Tolerance	VIP
(Constant)	41.9	2.2		<0.001	37.5	46.3	41.9	2.2
Sex (Female)	7.1	2.3	0.1	0.002	2.5	11.6	0.9	1.0
House type (No garden)	4.4	1.9	0.1	0.02	0.6	8.2	0.9	1.0
Academic course (Quarter)	5.0	2.2	0.1	0.02	0.5	9.5	0.9	1.0

*p* * value obtained with multiple linear regression.

## Data Availability

The data presented in this study are available on request from the corresponding author. The data are not publicly available due to privacy or ethical restrictions.
